# Supplementary material for: A clustering method for small scRNA-seq data based on subspace and weighted distance
Source: PeerJ. 2023 Jan 23;11:e14706. doi: 10.7717/peerj.14706 (PMC9879162; doi:10.7717/peerj.14706)
Supplement: Supplemental Information 2 [file peerj-11-14706-s002.docx]

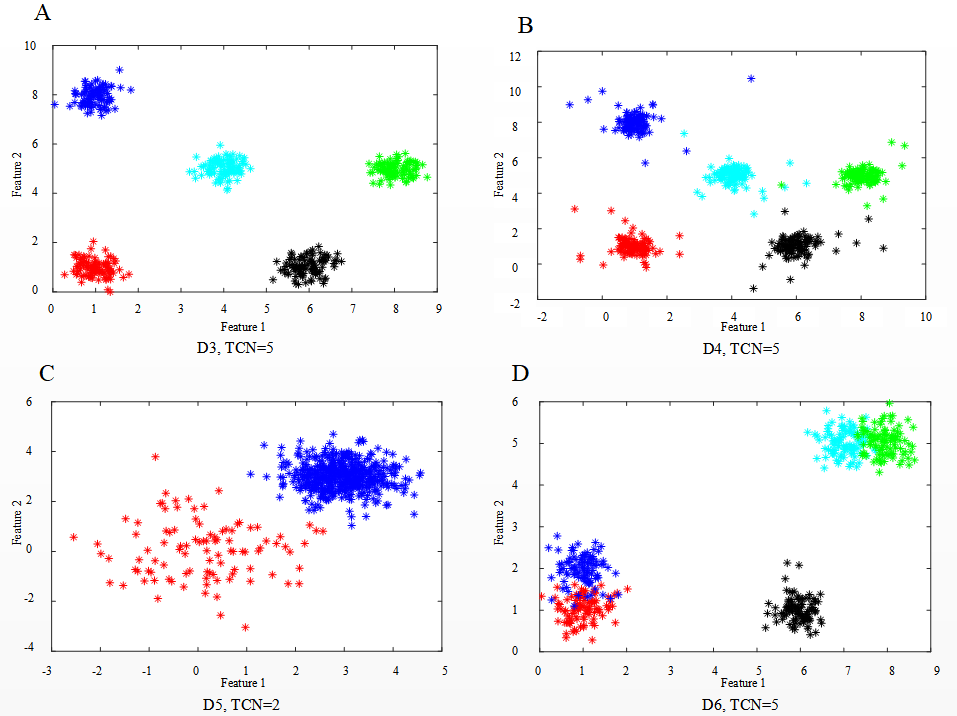


Figure S1 Four Gaussian datasets with different cluster properties(Liu et al., 2010; Hussain & Haris, 2019).

(A) D3 composing five “well-separated” clusters. (B) D4 is formed by adding 10% “noise” to dataset D3. (C) D5 is composed of two clusters with “different densities”. (D) D6 contains five clusters, in which two pairs of clusters can be regarded as two “subclusters”. *TCN* is the true cluster numbers.


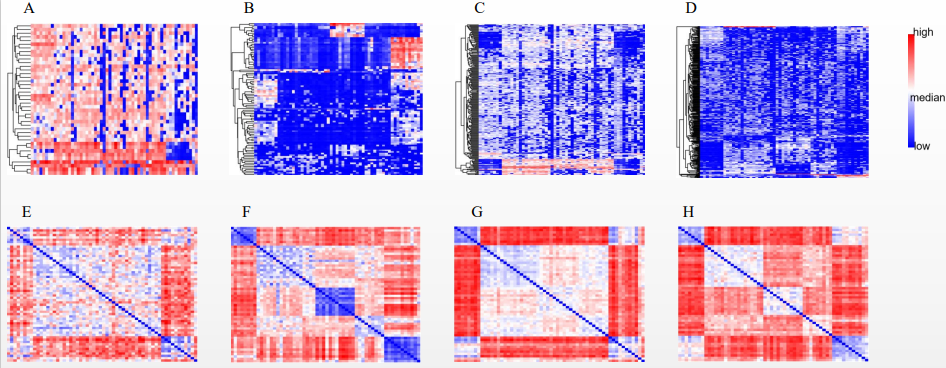


Figure S2：The four subspace expression heatmap and distance heatmap of Li

**References**

Liu, Y., Z. Li, X. Hui, X. Gao and J. Wu (2010). "Understanding of Internal Clustering Validation Measures." 2010 IEEE international conference on data mining. IEEE: 911-916

Hussain, S. F. and M. Haris (2019). "A k-means based co-clustering (kCC) algorithm for sparse, high dimensional data." Expert Systems with Applications **118**(15): 20-34.
